# Evaluation of MCF10A as a Reliable Model for Normal Human Mammary Epithelial Cells

**DOI:** 10.1371/journal.pone.0131285

**Published:** 2015-07-06

**Authors:** Ying Qu, Bingchen Han, Yi Yu, Weiwu Yao, Shikha Bose, Beth Y. Karlan, Armando E. Giuliano, Xiaojiang Cui

**Affiliations:** 1 Department of Surgery, Samuel Oschin Comprehensive Cancer Institute, Cedars-Sinai Medical Center, Los Angeles, California, United States of America; 2 Department of Obstetrics and Gynecology, Women’s Cancer Program, Samuel Oschin Comprehensive Cancer Institute, Cedars-Sinai Medical Center, Los Angeles, California, United States of America; 3 Department of Pathology, Samuel Oschin Comprehensive Cancer Institute, Cedars-Sinai Medical Center, Los Angeles, California, United States of America; 4 Department of Radiology, Shanghai Jiao Tong University Affiliated Sixth People’s Hospital, 600 Yishan Road, Shanghai, China; Georgetown University, UNITED STATES

## Abstract

Breast cancer is the most common cancer in women and a leading cause of cancer-related deaths for women worldwide. Various cell models have been developed to study breast cancer tumorigenesis, metastasis, and drug sensitivity. The MCF10A human mammary epithelial cell line is a widely used *in vitro* model for studying normal breast cell function and transformation. However, there is limited knowledge about whether MCF10A cells reliably represent normal human mammary cells. MCF10A cells were grown in monolayer, suspension (mammosphere culture), three-dimensional (3D) “on-top” Matrigel, 3D “cell-embedded” Matrigel, or mixed Matrigel/collagen I gel. Suspension culture was performed with the MammoCult medium and low-attachment culture plates. Cells grown in 3D culture were fixed and subjected to either immunofluorescence staining or embedding and sectioning followed by immunohistochemistry and immunofluorescence staining. Cells or slides were stained for protein markers commonly used to identify mammary progenitor and epithelial cells. MCF10A cells expressed markers representing luminal, basal, and progenitor phenotypes in two-dimensional (2D) culture. When grown in suspension culture, MCF10A cells showed low mammosphere-forming ability. Cells in mammospheres and 3D culture expressed both luminal and basal markers. Surprisingly, the acinar structure formed by MCF10A cells in 3D culture was positive for both basal markers and the milk proteins β-casein and α-lactalbumin. MCF10A cells exhibit a unique differentiated phenotype in 3D culture which may not exist or be rare in normal human breast tissue. Our results raise a question as to whether the commonly used MCF10A cell line is a suitable model for human mammary cell studies.

## Introduction

Breast cancer is the most common cancer in women and a leading cause of cancer-related deaths for women worldwide. To elucidate the mechanisms of breast cancer development and progression, different *in vitro* and *in vivo* models have been developed. Various mouse models have proven to be valuable in studying breast tumorigenesis, but these models each have limitations in fully recapitulating normal human breast and breast cancer development. *In vitro* culture of human mammary epithelial cells serves as a complementing approach. Conventional monolayer culture and more sophisticated three-dimensional (3D) culture systems have been widely used to study breast cell function, mammary gland morphogenesis, and breast cancer initiation. 3D culture, compared with 2D culture, better mimics *in vivo* conditions, and is thereby more desirable for investigating the cell behavior and function of normal and malignant cells. Matrigel, an ECM mixture isolated from Engelbreth-Holm-Swarm mouse sarcoma cells, provides the combination of extracellular matrix components that is similar to the *in vivo* microenvironment [1]. Collagen type I is the most abundant ECM component in the normal breast [2]. Use of Matrigel together with collagen I in 3D culture has been shown to be critical for generating functional acini and ducts *in vitro* [3–5].

The MCF10A human breast epithelial cell line is arguably the most commonly used normal breast cell model. These cells were derived from benign proliferative breast tissue and spontaneously immortalized without defined factors. They are not tumorigenic and do not express estrogen receptor [6]. Their known molecular characteristics include the depletion of the chromosomal locus containing the p16 and p14ARF genes, both of which are critical in regulating senescence, and amplification of the Myc gene [6]. When cultured on top of Matrigel, MCF10A cells are capable of forming acinus-like spheroids with a hollow lumen [7]. This structure is covered by basement membrane and formed by polarized and organized cells [3]. The 3D MCF10A model provides a useful tool for dissecting cell-cell interactions in mammary gland development, as well as for studying the effects of microenvironment on mammary cell function and the effects of different genetic or non-genetic modifications on mammary cell transformation.

Breast cancer is a heterogeneous disease and the heterogeneity of breast cancer cells may be inherited from their origins [8]. As such, proper models representing the sources of different breast cancer subtypes are desirable. To date, whether the MCF10A cell line is a suitable human mammary epithelial cell model has not been thoroughly evaluated. It has been shown that these cells exhibit a basal-like phenotype but share many features of mesenchymal cancer cell lines [9]. Here, we intend to address the suitability of MCF10A cells in modeling human mammary epithelial cells. For this purpose, we examined the expression of commonly used breast cell markers in MCF10A cells in well-established monolayer (2D), suspension (mammosphere culture), and different 3D culture systems. Our results show that MCF10A cells may not represent phenotypically normal luminal, basal, or progenitor/stem cells, thus questioning the relevance of MCF10A as a normal mammary epithelial model.

## Materials and Methods

### Human tissues

This study was approved by the Institutional Review Board (IRB) at Cedars-Sinai Medical Center. Normal human breast tissues were obtained from patients with written informed consent.

### Cell culture and medium

MCF10A cells (American Type Culture Collection, Manassas, VA) were cultured in DMEM/Ham's F-12 (GIBCO-Invitrogen, Carlsbad, CA) supplemented with 100 ng/ml cholera toxin, 20 ng/ml epidermal growth factor (EGF), 0.01 mg/ml insulin, 500 ng/ml hydrocortisone, and 5% chelex-treated horse serum. All of the growth factors were purchased from Sigma (St. Louis, MO, USA). MCF10A cells were subjected to no more than eight passages in culture when used in experiments. Human breast cancer cell lines MDA-MB-231 and MCF-7 were purchased from ATCC. Primary human mammary fibroblast cells (Lifeline Cell Technology, Frederick, MD) were used as negative controls for mammosphere formation and acinus growth in 3D culture.

### Mammosphere culture

Mammosphere culture was performed as previously described [10]. Cells were grown in the MammoCult medium (Stem Cell Technologies, Vancouver, Canada) supplemented with hydrocortisone, heparin, amphotericin B, and gentamicin (Sigma) and plated in ultra-low attachment plates (Corning Inc., Corning, NY). Cells were seeded in ultra-low attachment 96-well plates (Corning, Corning, NY, USA) at densities of 500 to 10,000 cells per well and grown for 14 days. The mammosphere formation rate was calculated as previously described [11] using the following equation: (number of mammospheres per well/number of cells seeded per well) × 100%.

### 3D culture

3D culture was performed as previously reported [12]. 3D “on-top” Matrigel culture was performed by making a layer of growth factor-reduced Matrigel (Sigma) in 24-well plates (120μl) or 96-well plates (15μl), which was then put back in a 37°C incubator for 30 min to solidify. After MCF10A cells were trypsinized and counted, 2.5×10^5^ cells in 250 μl and 5×10^4^ cells in 35 μl were seeded in 24-well and 96-well plates, respectively. Cells were allowed to attach for 30 min before adding an equal volume of medium with 10% Matrigel (v/v). 3D “cell-embedded” culture was performed by mixing trypsinized MCF10A cells in either Matrigel or mixed Matrigel/collagen I gel. The final concentration of collagen I in the mixed gel was 1mg/ml [13]. After gel was solidified, additional culture medium was added. The culture medium was changed every 3 days.

### Immunofluorescence staining

3D culture was fixed with 4% paraformaldehyde, washed with PBS, and embedded in Specimen Processing Gel (Histogel, American MasterTech Scientific, Lodi, CA). Samples were cooled and then processed by regular histological procedures. Cells were fixed with 4% paraformaldehyde, permeabilized with 0.5% Triton X-100 for 10 min, and blocked with 3% bovine serum albumin for 30 min at room temperature. After blocking, cells were incubated overnight at 4°C with primary antibodies (S1 Table). Alexa 594-conjugated secondary antibody (red, Molecular Probes, Eugene, OR) or an Alexa 488-conjugated secondary antibody (green, Molecular Probes) was used to visualize the staining. Following three washes with PBS, slides were mounted with the VECTASHIELD mounting medium (Vector Laboratories, Burlingame, CA). Prior to mounting, slides were incubated with 2 μM 4′,6-diamidino-2-phenylindole (DAPI) fluorescence (Molecular Probes) for 10 min at 37°C to stain the nuclei. The ER^+^ luminal breast cancer cell line MCF-7, the triple-negative breast cancer cell line MDA-MB-231, and normal human mammary gland tissues were used as controls for immunofluorescence staining. The fluorescence images were taken using an Olympus IX51 fluorescence microscope (Olympus, Center Valley, PA).

### Immunohistochemical staining

Suspension or 3D culture was fixed with 4% phosphate-buffered formalin and embedded in paraffin. Blocks were cut into 4-μm thick slides for the following staining. Slides were deparaffinized and rehydrated by xylene and gradient ethanol, respectively. An antigen retrieval method using microwave pretreatment and 0.01 M sodium citrate buffer (PH6) was used for all antibodies (Vector laboratories, Burlingame, CA). The signal was visualized using the VECTASTAIN ABC Systems (Vector laboratories). Counterstaining was performed with Mayer’s hematoxylin (Sigma). Staining was visualized and pictures were captured using an Olympus BX51 light microscope (Olympus).

### Statistical analysis

Values represent mean ± standard deviation (SD) of samples measured in triplicate. Data present three independent experiments. Quantitative data were analyzed using the Student’s t test and two-tailed distribution.

## Results

### The morphologies of MCF10A cells in different culture conditions

**Fig 1:**
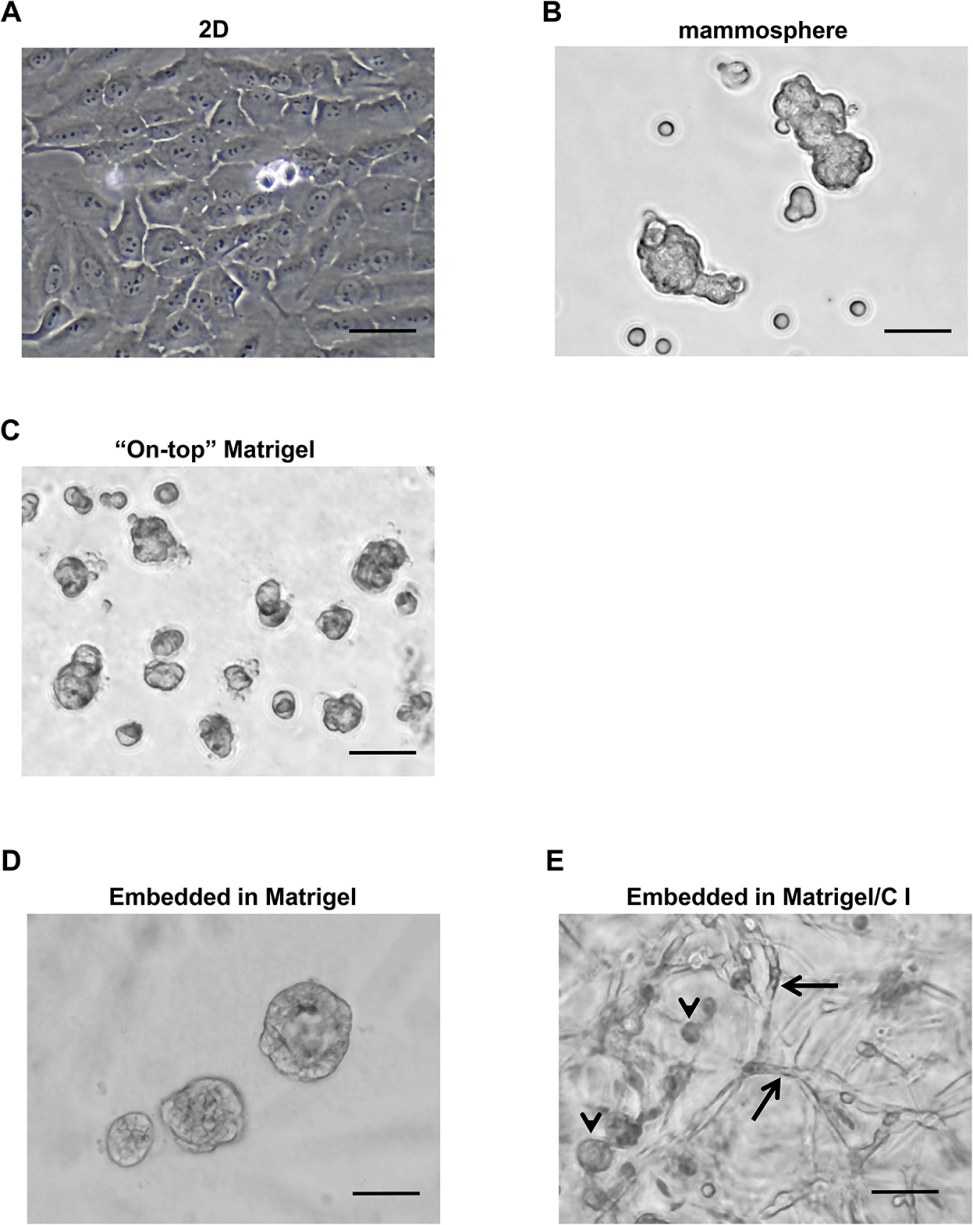
MCF10A cells in different culture systems. **(A)** MCF10A cells in monolayer 2D culture. **(B)** MCF10A cells were cultured in ultra-low attachment 96-well plates with the MammoCult medium. **(C)** MCF10A cells in “on-top” of Matrigel. **(D)** MCF10A cells embedded in Matrigel. **(E)** MCF10A cells embedded in mixed Matrigel/collagen I gel. Spheres and branches in 3D culture were indicated by black arrow heads and black arrows, respectively. Bars: 100μm. Original magnification: ×200.

We first examined the morphologies of MCF10A cells in different culture systems. Regular 2D culture of MCF10A cells yielded a cuboidal epithelial morphology (Fig 1A). When using suspension culture to enrich the stem/progenitor cell populations, MCF10A cells formed mammospheres (Fig 1B). In 3D culture of “on-top” Matrigel, MCF10A cells produced spheroid structures (Fig 1C), as previously discovered [14]. When embedded in Matrigel, the majority of MCF10A cells also formed spheroid structures (Fig 1D). We also tested MCF10A cell growth in mixed Matrigel/collagen I gel. Collagen I has been used in the *in vitro* 3D culture system because its mechanical tension on cells facilitates their 3D structure formation. In addition, the mixture of Matrigel and collagen I was found to create a better microenvironment for breast epithelial cells to grow branches as well as acini [13]. As expected, spheroid (Fig 1E, black arrow heads) and branch structures (Fig 1E, black arrows) were observed when the cells were embedded in mixed Matrigel/collagen I gel. These data suggest that MCF10A cells can form distinct structures in different culture conditions.

### MCF10A cells express basal/myoepithelial and luminal markers in 2D culture

**Fig 2:**
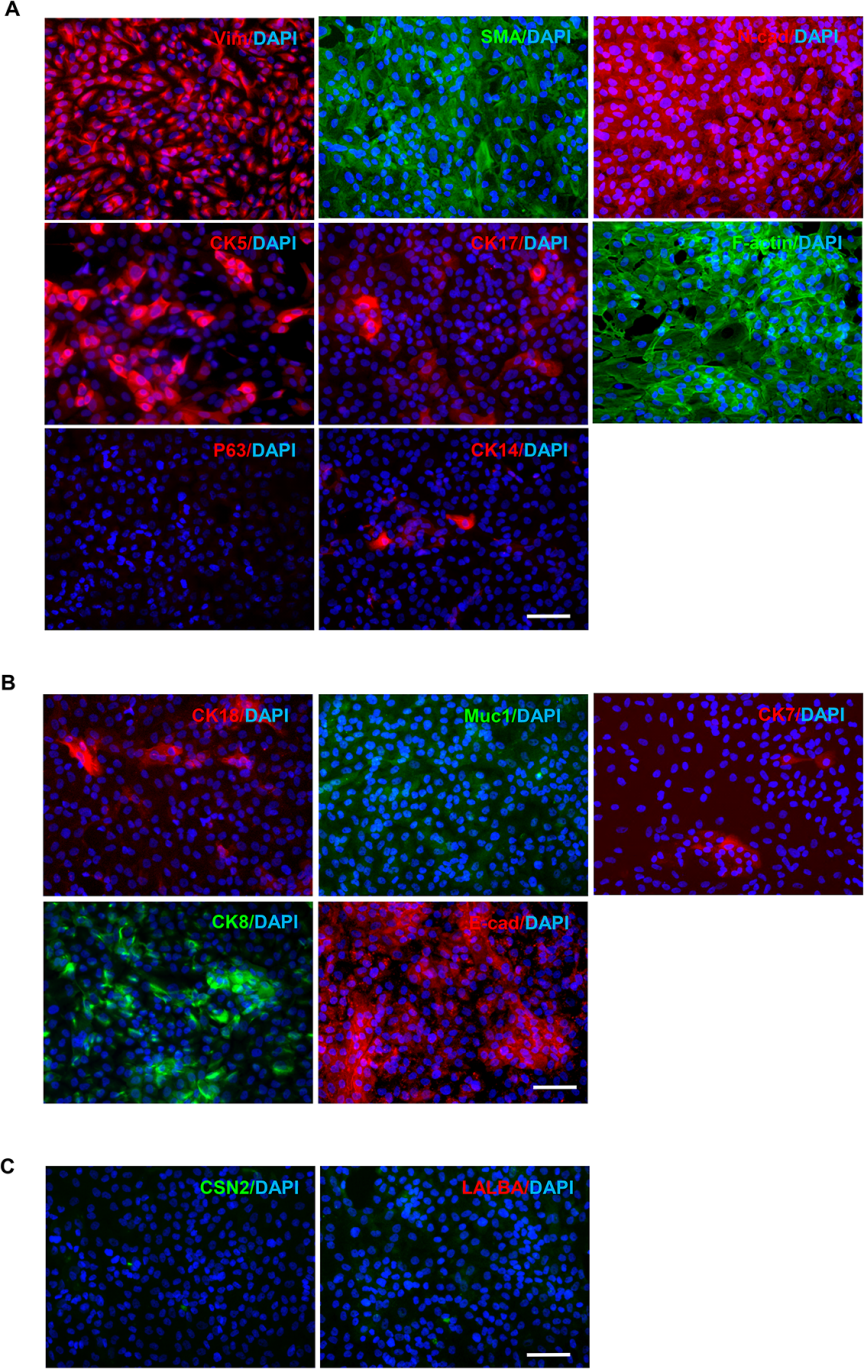
Immunofluorescence staining of the mammary cell markers in MCF10A cells in 2D culture. MCF10A cells grown in monolayer were fixed and subjected to immunofluorescence staining using the antibodies against basal cell **(A)**, luminal cell **(B)**, and breast tissue markers **(C)**. Secondary antibodies were either AF488 (green)—or AF594 (red)-conjugated. DAPI was used for nuclear staining. Bars: 50μm. Original magnification: ×200.

Cytokeratin (CK) expression patterns are commonly used to identify mammary epithelial cells. Myoepithelial cells typically express CK5/6, CK14, and CK17 [15] as well as other markers such as P63 [16], vimentin [17], and alpha smoothen actin (SMA) [18], whereas luminal cells are characterized by CK8, CK18 [19], and CK7 [20, 21], as well as other markers such as Mucin1 (Muc1) [22] and E-cadherin (E-cad) [23]. Hence, we used immunofluorescence staining to profile the expression of these basal and luminal markers in MCF10A cells As shown in Fig 2A and S2 Table, MCF10A cells strongly expressed vimentin, alpha smooth muscle Actin (SMA), and the basal-cadherin N-cadherin [24], suggesting a basal origin for MCF10A. Around 50% of cells displayed strong staining of CK5, whereas around 10% of cells expressed moderate levels of CK17. Phalloidin staining (F-actin) indicated stress fiber formation in MCF10A cells. Surprisingly, MCF10A cells exhibited completely negative staining of P63 and less than 1% of cells were positive for CK14.

As for luminal markers, less than 10% of cells expressed CK18, CK7, and mucin 1 (Muc1) (Fig 2B). The proportion of CK8-positive staining was 50%. E-cad was abundantly expressed and localized in both cytoplasm and cell membrane (Fig 2B). We also confirmed that MCF10A cells did not express estrogen receptor (ER) and progesterone receptor (PR) (data not shown) as previously reported [6]. As a control, MCF-7 luminal breast cancer cells showed negative staining of basal markers (P63, CK14, Vim, CK5, CK17, N-cad) but positive staining of luminal markers CK18, CK8, E-cad, CK7, Muc1, ER, and PR (S1A Fig). In contrast, MDA-MB-231 basal breast cancer cells displayed basal markers CK14 and vimentin, but not the luminal markers CK8 and CK18 (S1B Fig).

We next assessed the expression of β-casein (CSN2) and α-lactalbumin (LALBA), which were reported to be specifically expressed in breast tissue [25–27]. As presented in Fig 2C, MCF10A cells showed negative staining of CSN2 and LALBA in the cytoplasm. Collectively, these data suggest that MCF10A cells in 2D culture exhibit a basal-like phenotype but concomitantly express luminal markers.

### MCF-10A cells express stem/progenitor markers in monolayer culture

**Fig 3:**
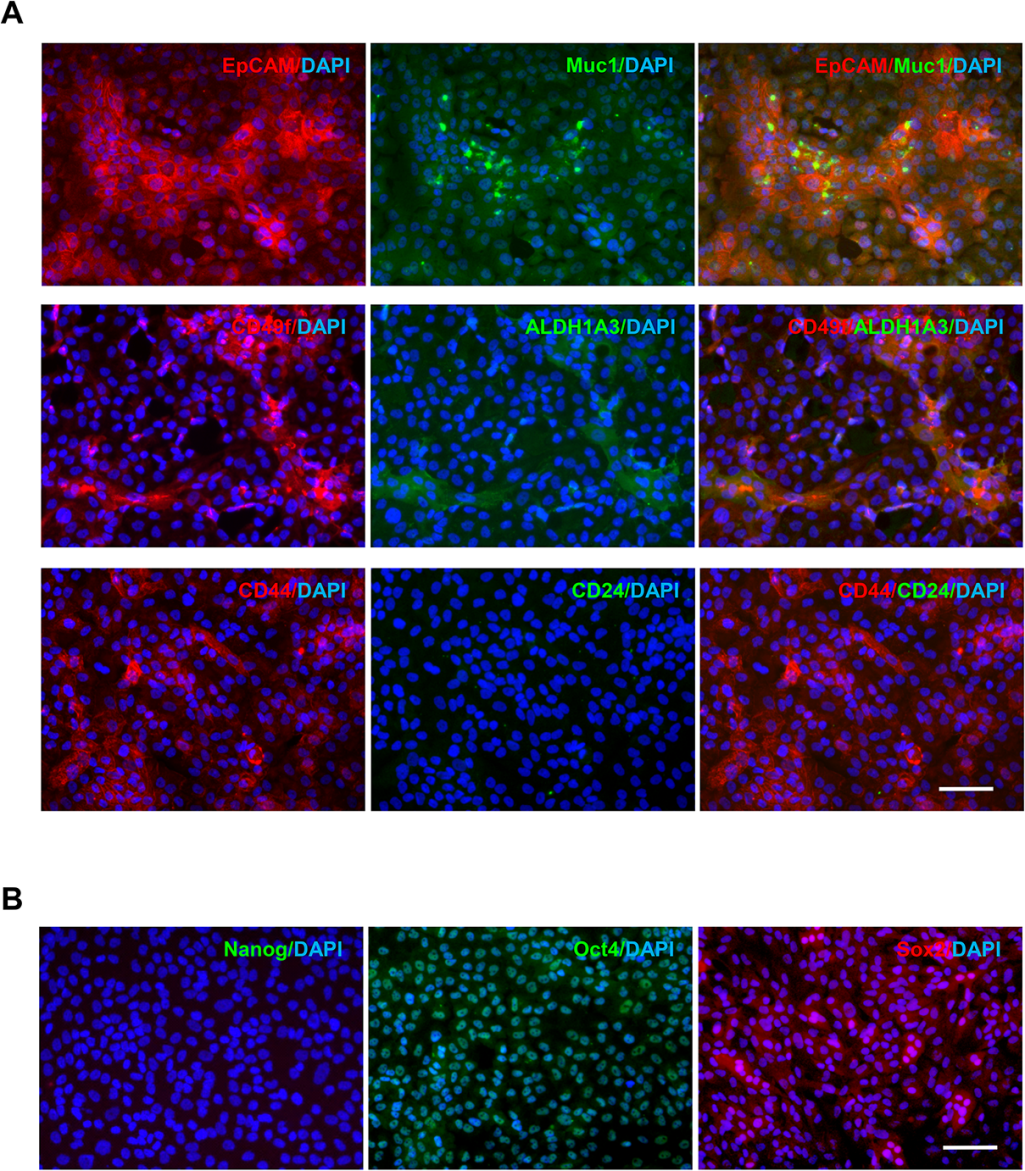
Immunofluorescence staining of the stem-like cell populations in MCF10A cells in 2D culture. MCF10A cells grown in monolayer were fixed and stained using the antibodies against reported mammary stem/progenitor **(A)** and stem-like **(B)** markers. Secondary antibodies were either AF488 (green)—or AF594 (red)—conjugated. DAPI was used for nuclear staining. Bars: 50μm. Original magnification: ×200.

We next asked whether MCF10A cells express reported breast stem/progenitor cell markers. It was demonstrated that EpCAM^+^/Muc1^−^ epithelial cells within the luminal epithelial lineage may function as precursor cells of the terminal duct lobular units in the human breast [28]. Studies also showed that breast stem/progenitor cells may possess the EpCAM^+^/CD49f^+^ [8, 29], Aldehyde dehydrogenase (ALDH1) ^high^ [30], or CD44^+^/CD24^-^ [31] phenotype. In particular, ALDH activity is widely used to identify stem/progenitor cells [30], and this activity can be attributed to ALDH1A3 in stem cells [32]. Using immunofluorescence staining, we found that a high percentage of MCF10A cells exhibited the EpCAM^+^/Muc1^-^, ALDH1A3^+^/CD49f^+^, or CD44^+^/CD24^-^ phenotype (Fig 3A and S3 Table). Next, we examined the expression of the stem cell-associated transcription factors Sox-2, Nanog, and Oct4 [33] in MCF10A cells. As presented in Fig 3B, MCF10A cells did not express Nanog but expressed high levels of Oct4 and Sox2. These data suggest that MCF10A cells contain sub-populations expressing stem/progenitor markers.

### Mammosphere formation of MCF10A cells

**Fig 4:**
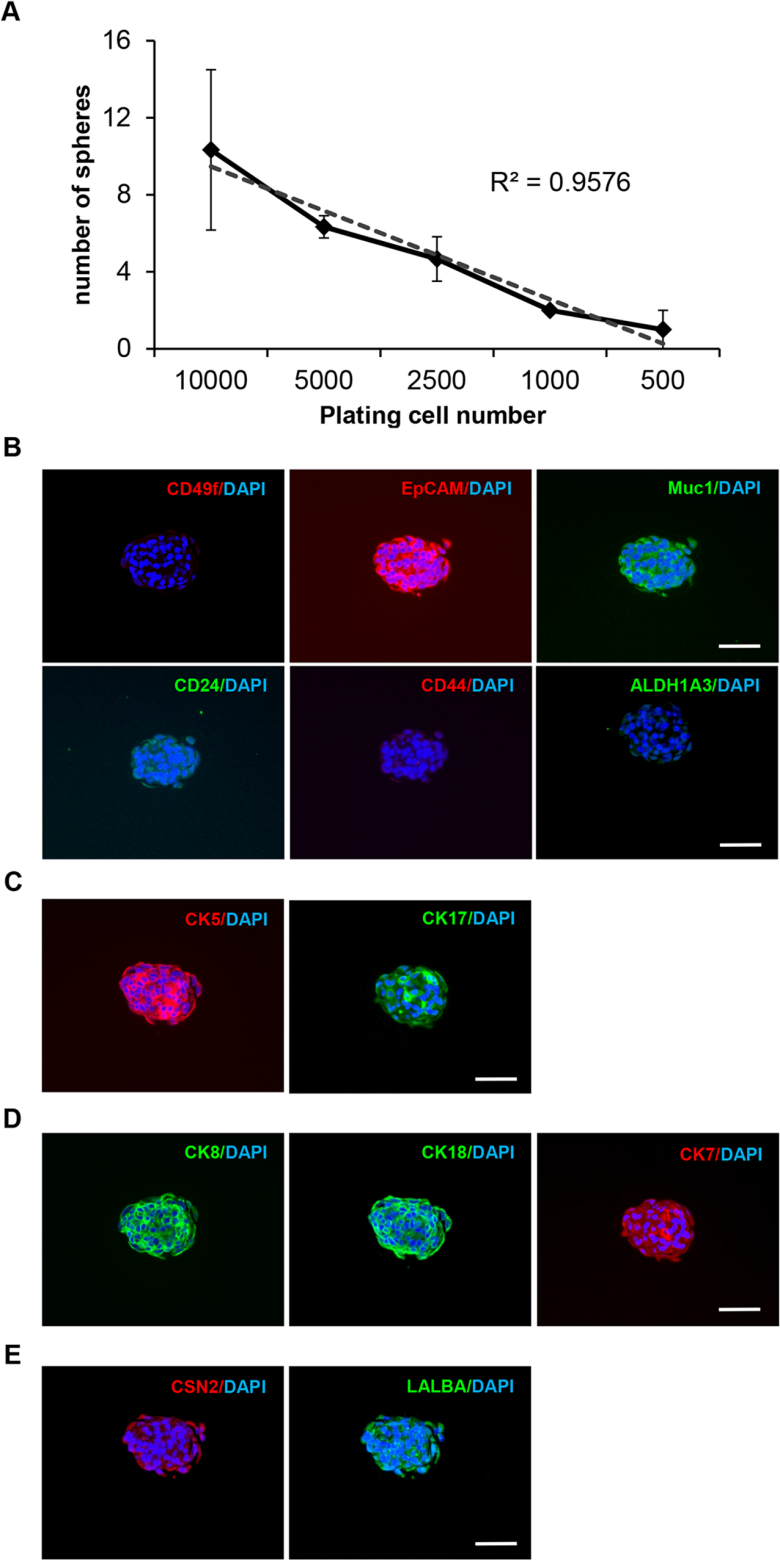
Mammosphere culture of MCF10A cells. **(A)** MCF10A cells subjected to serial dilution and mammosphere formation assays. Each group was triplicated and the number of spheres was counted and plotted as mean ± SD. The mammospheres formed by MCF10A cells were fixed and embedded for histological analysis. Serial sections were cut for immunofluorescence staining against stem/progenitor markers **(B)**, basal markers **(C)**, luminal markers **(D)**, and breast tissue markers **(E)**. Secondary antibodies were either AF488 (green)—or AF594 (red)—conjugated. DAPI was used for nuclear staining. Bars: 50μm. Original magnification: ×200.

Next, we characterized the self-renewal ability of MCF10A cells by using a mammosphere culture method, which has been used to quantify both stem cell/early progenitor activity and self-renewal in the normal mammary tissue [11]. To test the stem/progenitor property of MCF10A cells, we performed mammosphere assays using a serial dilution to seed 500 to 10,000 cells per well. The number of formed spheres after 14 days was positively correlated with the initial cell number (R^2^ = 0.9576) (Fig 4A). Of note, the mammosphere formation rate of MCF10A cells was (0.16 ± 0.04) %. These data suggest that MCF10A cells have a low ability to form mammosphere although stem/progenitor markers are highly expressed. However, when we sorted MCF10A cells by using common stem cell markers (S1 File) such as CD49f, ALDH1A3, and CD44, the CD44^+^/CD24^-^ and CD49f^+^ populations were found to yield more mammospheres than the CD44^+^/CD24^+^ and CD49f^-^ populations, respectively, whereas the ALDH1A3^+^ and ALDH1A3^-^ populations did not show a difference (S2 Fig). Our data suggest that these individual markers may be sufficient for distinguishing the whole stem-like population in MCF10A cells. Alternatively, as suggested by recent reports [34, 35], these commonly used stem cell markers may not represent stem-like cells in the MCF10A cell line.

Because mammosphere culture has been used to enrich cells with stem-like characteristics [36], we examined the expression of differentiation and stem/progenitor markers in MCF10A mammospheres using immunofluorescence staining of continuous thin sections from the fixed sphere collection. Interestingly, loss of stem/progenitor markers and gain of differentiation markers were found in mammosphere-forming cells (Fig 4B–4E). These cells exhibited the CD49f^-^, EpCAM^+^, Muc1^+^, CD24^+^, CD44^-^, and ALDH1A3^-^ phenotypes (Fig 4B), and expressed the basal markers CK5 and CK17 (Fig 4C), luminal markers CK8, CK18 and CK7 (Fig 4D), and breast specific markers CSN2 and LALBA (Fig 4E). It merits mentioning that CK18, CK7, CSN2, and LALBA, barely detectable or undetected in MCF10A cells in 2D culture, were abundantly expressed in mammospheres (S4 Table). These data suggest that MCF10A mammospheres are composed of differentiated luminal and basal cell populations.

### MCF10A cells in 3D Matrigel culture

We next investigated the expression profiles of those markers in MCF10A cells in 3D culture. To do so, we first used the “on-top” Matrigel method by plating single MCF10A cells on a layer of pre-solidified thick Matrigel followed by adding 5% of Matrigel in the medium [12]. We also embedded MCF10A cells in Matrigel to grow 3D structure. In agreement with previous studies, MCF10A cell formed well-characterized polarized acini in both “on-top” and “cell-embedded” Matrigel culture (data not shown).

**Fig 5:**
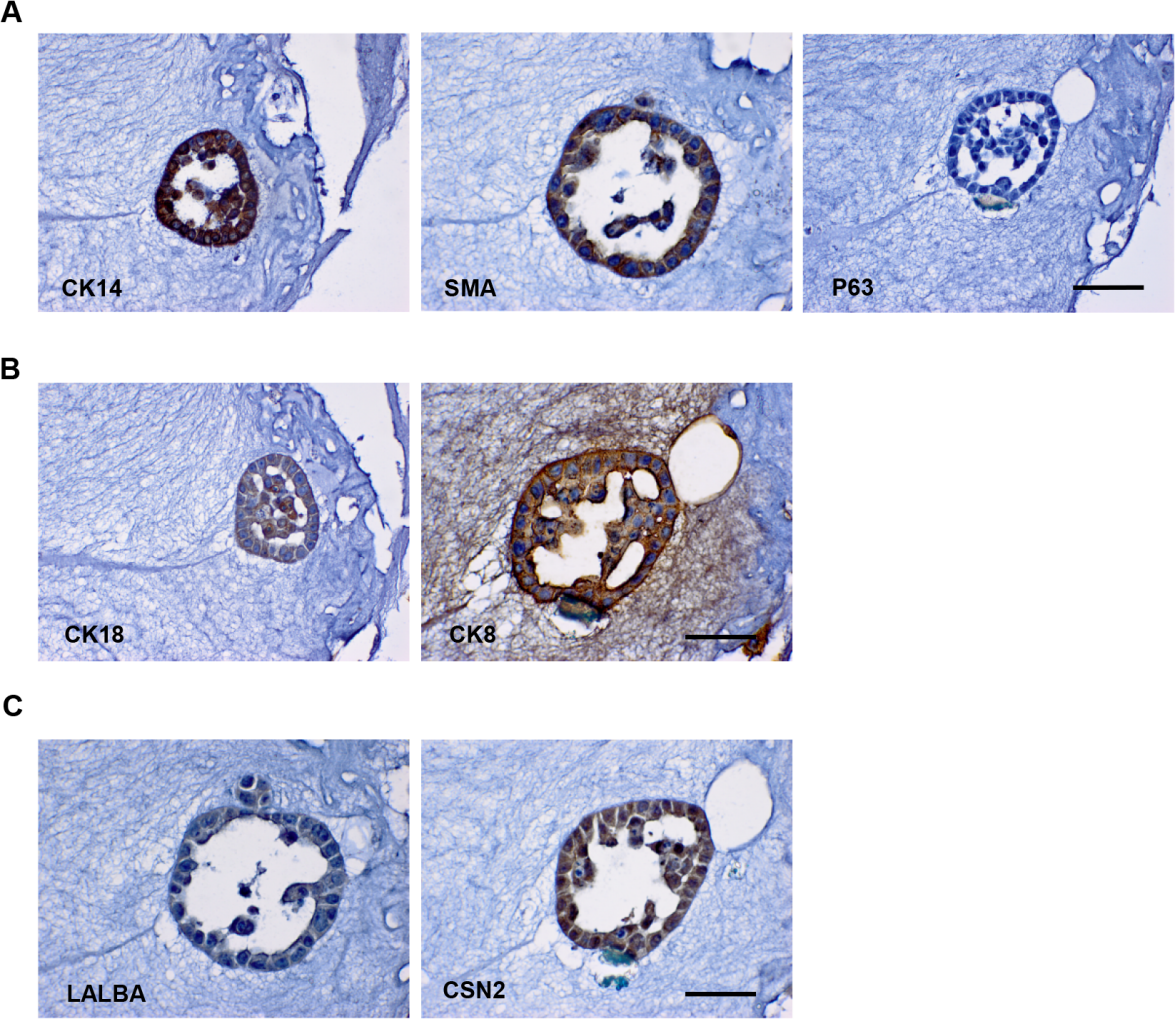
MCF10A cells in “on-top” of Matrigel. MCF10A cells were cultured in “on-top” of Matrigel for 14 days. 3D cultures were fixed, embedded and cut into sections. Immunohistochemistry staining of basal markers **(B)**, luminal markers **(C)**, and breast tissue-specific markers **(D)** were performed. Bars: 50μm. Original magnification: ×600.

We then tested the expression of markers using continuous thin sections from fixed acini. Similar to mammospheres, MCF10A spheres in “on-top” Matrigel did not express stem-like markers such as CD49f (data not shown) but expressed the basal markers CK14 and SMA (Fig 5A). However, P63 was not detected (Fig 5A). These cells also expressed luminal CK18 and CK8 (Fig 5B) as well as breast specific markers CSN2 and LALBA (Fig 5C), suggesting that luminal-, basal- and breast tissue-specific- markers were co-expressed in the cells. Of note, immunofluorescence staining of mammosphere thin sections showed that MCF10A cells did not express the stem/progenitor markers CD49f, CD44, and ALDH1A3 at day 7 and day 10 of Matrigel culture (S3 Fig), suggesting that Matrigel may induce the loss of stem/progenitor marker expression in MCF10A cells. To further test whether these spheres were positive for both luminal- and basal-markers, we performed immunofluorescence co-staining. As shown in S4A Fig, both CK18 and CK14 were positive in the acinar structure and thus co-expressed in the same cells. In contrast, the majority of luminal cells were CK18^+^ and basal cells were CK14^+^ in the normal human mammary gland tissue (S4B Fig). More importantly, cells in the duct did not co-express CK14 and CSN2 (S4C Fig). MCF10A cells embedded in Matrigel showed similar results as the “on-top” culture (data not shown). We also found that co-culture of mammary fibroblast cells did not alter MCF10A cell differentiation profiles or luminal, basal, breast tissue marker expression pattern in Matrigel 3D culture (data not shown). These data suggest that MCF10A cells are able to form a polarized spheroid structure but may not represent normal human breast epithelial cells or may represent a rare mammary epithelial cell type.

### MCF10A cells in mixed Matrigel/collagen I culture

**Fig 6:**
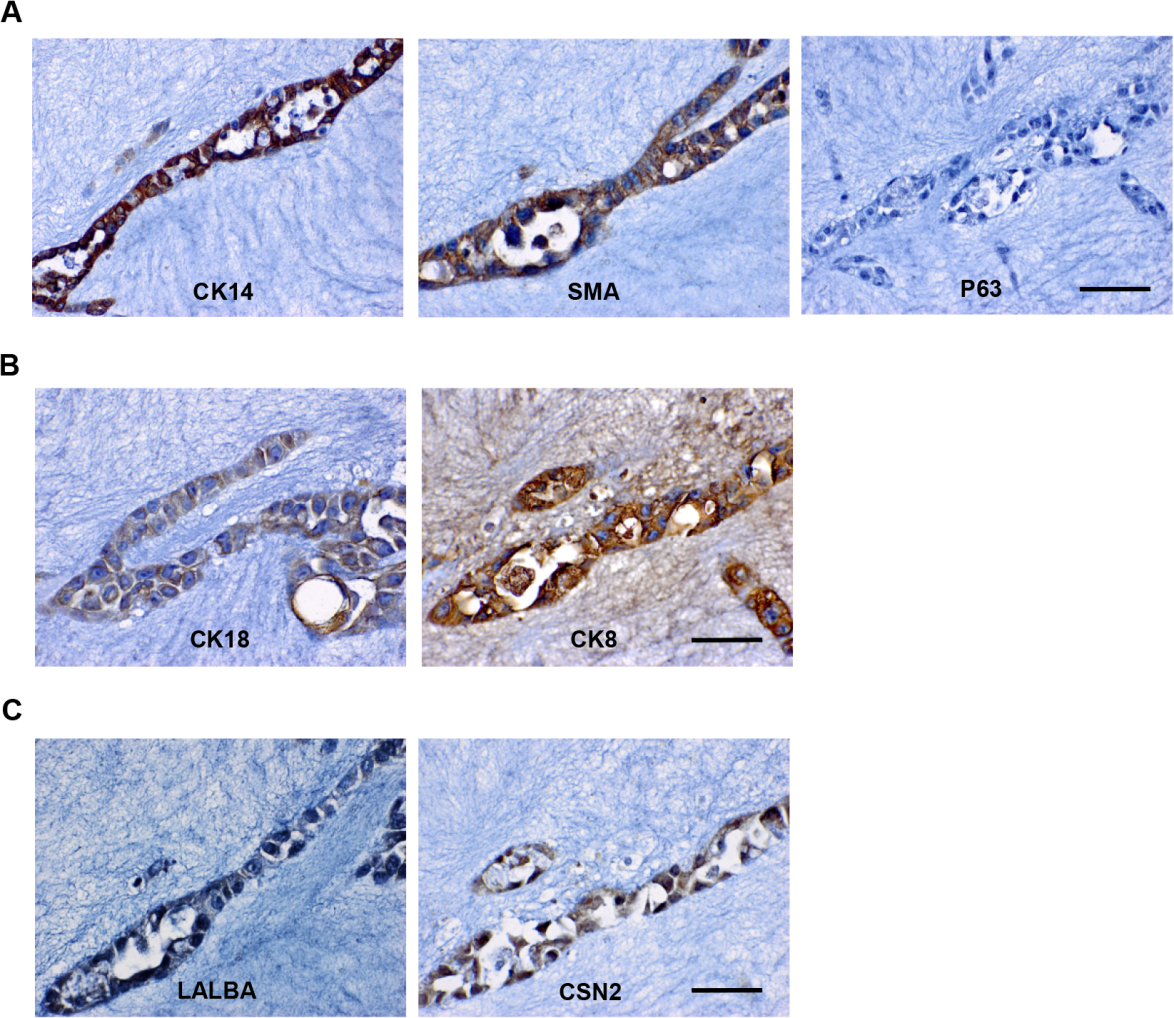
MCF10A cells embedded in mixed Matrigel/collagen I gel. Immunohistochemistry staining of basal markers **(A)**, luminal markers **(B)**, and breast tissue-specific markers **(C)** were performed in 3D-cultured MCF10A cells. Bars: 50μm. Original magnification: ×600.

When embedded in the mixed gel, MCF10A cells formed acini characterized as spherical mono-layers of cells that enclose a central lumen and branches formed by elongated cells. As expected, MCF10A cells-formed branches expressed basal markers CK14 and SMA, but not P63 (Fig 6A). They also expressed luminal markers CK18 and CK8 (Fig 6B) as well as the breast tissue-specific markers CSN2 and LALBA (Fig 6C). The spherical structures showed the same marker expression (data not shown). These observations suggest that MCF10A cells in mixed Matrigel/collagen I gel also do not possess a common marker profile reflecting those of known human mammary epithelial cell types.

## Discussion

MCF10A cells are widely used non-malignant breast epithelial cells. They exhibit some features of normal breast epithelium, including lack of anchorage-independent growth and dependence on growth factors and hormones for proliferation and survival [6]. They can form acinar structures in 3D culture [37]. These traits render these cells a good model to analyze the effects of oncogenes [38–40]. However, the identity of MCF10A cells is not well understood, although MCF10A cells were previously classified as a basal-type non-transformed human mammary breast epithelial cell line based on gene expression profiling [9, 41]. In this study, we examined a series of makers including luminal and myoepithelial cells, milk proteins, and stem-like cells in MCF10A cells in 2D and 3D culture. Our results challenge MCF10A cells as a representative model for normal mammary cells.

### The characteristics of MCF10A cells in 2D culture

In our study, we found that MCF10A cells in 2D culture possess distinct cell populations. MCF10A cells only expressed CK5 and CK17, rarely CK14 and no P63. Notably, these cells ubiquitously expressed high levels of SMA and vimentin, sharing the features of myoepithelial cells. Although MCF10A cells abundantly express CK8, the expression levels of other luminal markers such as CK7, Muc1, and CK18 were either low or undetectable. In addition, these cells express both the epithelial cell-cell junction protein E-cadherin and the basal cell junction protein N-cadherin. It has been documented that the *in vitro* culture of mammary epithelial cells tends to create basal-like cell lines [8]. A recent study using a panel of luminal and myoepithelial lineage markers classifies cells into luminal, basal, and mesenchymal types [8]. MCF10A may be a basal-type breast epithelial cell line showing luminal features or a luminal-type cell line undergoing epithelial-to-mesenchymal transition (EMT) [42].

### Stem-like properties of MCF10A cells

We found that MCF10A cells express stem cell-like markers, suggesting their stem/progenitor-like properties. However, the mammosphere formation rate in MCF10A cells was low compared with the high percentages of stem/progenitor marker-positive cells. This paradoxical finding may be because the ability to form mammospheres is not necessarily correlated with the expression of stem/progenitor markers [34]. Sara and her colleagues discovered that the expression of different stem/progenitor markers may actually indicate different molecular subtypes [43]. A recent finding also suggested that the stem/progenitor cells identified by different markers or assays might represent distinct populations of cells with distinct stem/progenitor properties [31]. Studies have shown that the positivity of some stem-like markers in cells does not correlate with their ability to form mammospheres [32, 44]. Of note, we found that mammospheres comprise both luminal and myoepithelial cells with diminished expression of stem/progenitor-cell markers. Hence, whether MCF10A mammospheres enrich stem/progenitor cells is questionable.

### The identity of MCF10A cells in 3D structure

It is known that MCF10A cells are able to form growth-arrested and polarized acini in Matrigel culture. When collagen I was included, MCF10A cells formed acini as well as branches as previously reported [13, 45]. These 3D structures did not show positivity for stem/progenitor marker staining. Nevertheless, we observed a seemingly paradoxical phenotype of MCF10A cells when grown in 3D condition. First, we found co-expression of luminal and basal/myoepithelial markers such as CK14/SMA and CK18/CK8 in acini and branches formed in 3D cultures. As some cells in the luminal layer in the normal human mammary gland also express myoepithelial cell markers [20], MCF10A cells may only represent that particular population. However, it is worth mentioning that it is still not clear whether those rare cells co-express luminal and basal markers or if they are basal cells in the luminal epithelium. Second, MCF10A cells in 3D culture were found to homogeneously express CSN2 and LALBA, two differentiated luminal epithelial cell markers in human breast. Functionally and structurally, 3D-cultured MCF10A cells shared some features of human luminal mammary epithelial cells. However, both basal markers and milk proteins were found to be co-localized in branches and acini, which have not been reported in the normal mammary gland. Thus, MCF10A cells-formed acini may not reliably represent human mammary gland structure. Our findings have shown an variable phenotype of MCF10A cells under different culture conditions. Further studies are needed to confirm whether MCF10A cells serve as an appropriate model for normal mammary epithelial cells.

## Conclusions

In summary, our studies demonstrate that MCF10A cells exhibit a basal-like phenotype but express luminal and stem-like markers in 2D culture and present an unique epithelial-cell marker expression profile in 3D culture that have not been observed in mammary gland tissues. As MCF10A cells show different profiles of commonly used markers in both 2D and 3D culture compared with normal mammary tissue, whether MCF10A cells represent a suitable model for human mammary epithelial cells warrants further investigation.

## Supporting Information

S1 File Supplementary Materials and Methods. Immunostaining and FACS sorting. Approximately 5×10^6^ cells were suspended in FACS buffer (1×PBS, 1% BSA) and incubated with antibodies at 4°C for 30 min. The antibodies were CD24-FITC, CD44-PE, CD49f-FITC and EpCAM-PE (BD Biosciences). Cells were washed three times and filtered to remove extra antibodies and cell clusters. All samples were analyzed and sorted using Aria III Cell sorter (BD Biosciences). ALDH activity assay. Detection of ALDH activity was performed using the ALDEFLUOR Assay Kit (StemCell Technologies) according to the manufacturer’s instruction. Cells were analyzed and sorted using Aria III Cell sorter (BD Biosciences).
(DOCX)

S1 Fig Immunofluorescence staining of luminal and basal markers in luminal breast cancer cell line MCF-7 (A) and basal breast cancer cell line MDA-MB-231 (B). Secondary antibodies were either AF488 (green)—or AF594 (red)—conjugated. DAPI was used for nuclear staining. Bars: 100μm. Original magnification: ×200.
(TIF)

S2 Fig Mammosphere formation of FACS-sorted MCF10A sub-populations. MCF10A cells were stained with CD44-PE/CD24-FITC, CD49f-FITC/EpCAM-PE, or ALDEFLUOR. The positive and negative cells were isolated by FACS sorting. The sorted MCF10A cells were subjected to a serial dilution and mammosphere formation assays. Each group was performed in triplicate and the number of spheres was counted and plotted as mean ± SD. #: p<0.001. *: p<0.05.
(TIF)

S3 Fig Immunofluorescence staining of stem/progenitor markers in MCF10A cells cultured “on-top” of Matrigel. MCF10A cells were cultured using “on-top” of Matrigel method. 3D cultures were fixed, embedded and cut into sections. Expression of stem/progenitor markers were analyzed by immunofluorescence staining. Secondary antibodies were either AF488 (green)—or AF594 (red)—conjugated. DAPI was used for nuclear staining. Bars: 50μm. Original magnification: ×200.
(TIF)

S4 Fig Immunofluorescence staining of markers in MCF10A cells cultured “on-top” of Matrigel and normal human breast tissue. **(A)** Expression of CK18 (luminal) and CK14 (basal) in MCF10A formed acini. Enlarged views (white squares) of the indicated area (white dash squares) are shown. **(B)** CK14/CK18 double staining showed CK14+ basal layer and CK18+ luminal layer. **(C)** CSN2/CK14 double staining in normal human breast tissue. Secondary antibodies were either AF488 (green)—or AF594 (red)—conjugated. DAPI was used for nuclear staining. Bars: 100μm. Original magnification: ×200.
(TIF)

S1 Table The primary antibodies used in this study. (DOCX)

S2 Table Percentage of MCF10A cells expressing basal, luminal or breast-specific markers in 2D culture. Data represent the average positive cell percentage calculated from 10 viewing fields (original magnification, ×200).
(DOCX)

S3 Table Percentage of MCF10A cells expressing stem/progenitor markers in 2D culture. Data represent the average positive cell percentage calculated from 10 viewing fields (original magnification, ×200).
(DOCX)

S4 Table Percentage of MCF10A cells expressing indicated markers in mammospheres. Data represent the average positive cell percentage calculated from 10 viewing field in a thin section (original magnification, ×200).
(DOCX)
