# Supplementary material for: Evaluation of MCF10A as a Reliable Model for Normal Human Mammary Epithelial Cells
Source: PLoS One. 2015 Jul 6;10(7):e0131285. doi: 10.1371/journal.pone.0131285 (PMC4493126; doi:10.1371/journal.pone.0131285)
Supplement: S1 File — Immunostaining and FACS sorting. Approximately 5×10^6^ cells were suspended in FACS buffer (1×PBS, 1% BSA) and incubated with antibodies at 4°C for 30 min. The antibodies were CD24-FITC, CD44-PE, CD49f-FITC and EpCAM-PE (BD Biosciences). Cells were washed three times and filtered to remove extra antibodies and cell clusters. All samples were analyzed and sorted using Aria III Cell sorter (BD Biosciences). ALDH activity assay. Detection of ALDH activity was performed using the ALDEFLUOR Assay Kit (StemCell Technologies) according to the manufacturer’s instruction. Cells were analyzed and sorted using Aria III Cell sorter (BD Biosciences). (DOCX) [file pone.0131285.s001.docx]

**Supplementary Materials and Methods**

**Immunostaining and FACS sorting**

Approximately 5×10^6^ cells were suspended in FACS buffer (1×PBS, 1% BSA) and incubated with antibodies at 4°C for 30 min. The antibodies were CD24-FITC, CD44-PE, CD49f-FITC and EpCAM-PE (BD Biosciences). Cells were washed three times and filtered to remove extra antibodies and cell clusters. All samples were analyzed and sorted using Aria™ III Cell sorter (BD Biosciences).

**ALDH activity assay**

Detection of ALDH activity was performed using the ALDEFLUOR Assay Kit (StemCell Technologies) according to the manufacturer’s instruction. Cells were analyzed and sorted using Aria™ III Cell sorter (BD Biosciences).
