# Supplementary figures and images for: Evaluation of MCF10A as a Reliable Model for Normal Human Mammary Epithelial Cells
Source: PLoS One. 2015 Jul 6;10(7):e0131285. doi: 10.1371/journal.pone.0131285 (PMC4493126; doi:10.1371/journal.pone.0131285)

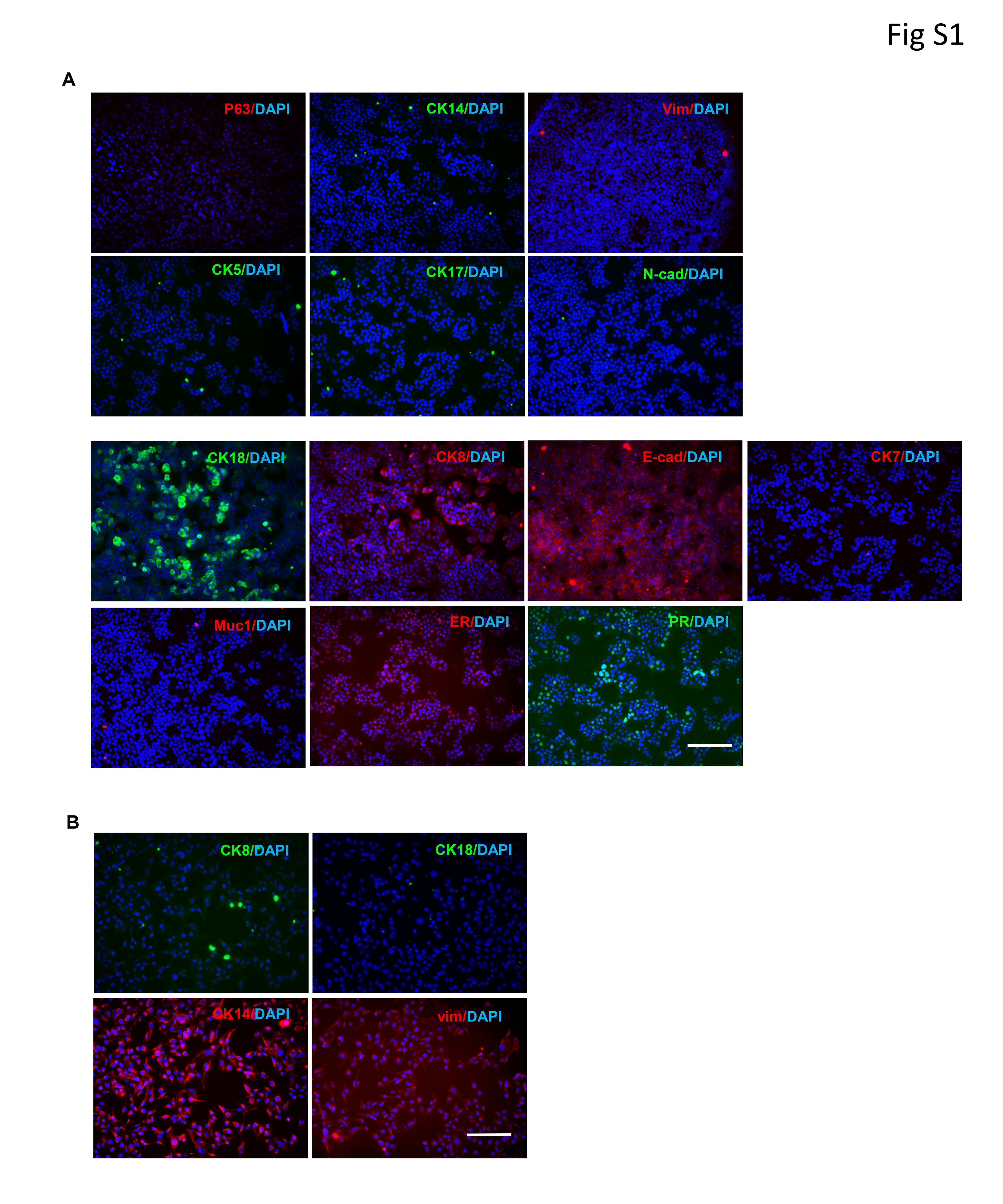

Supplement: S1 Fig — Secondary antibodies were either AF488 (green)—or AF594 (red)—conjugated. DAPI was used for nuclear staining. Bars: 100μm. Original magnification: ×200. (TIF) [file pone.0131285.s002.tif]

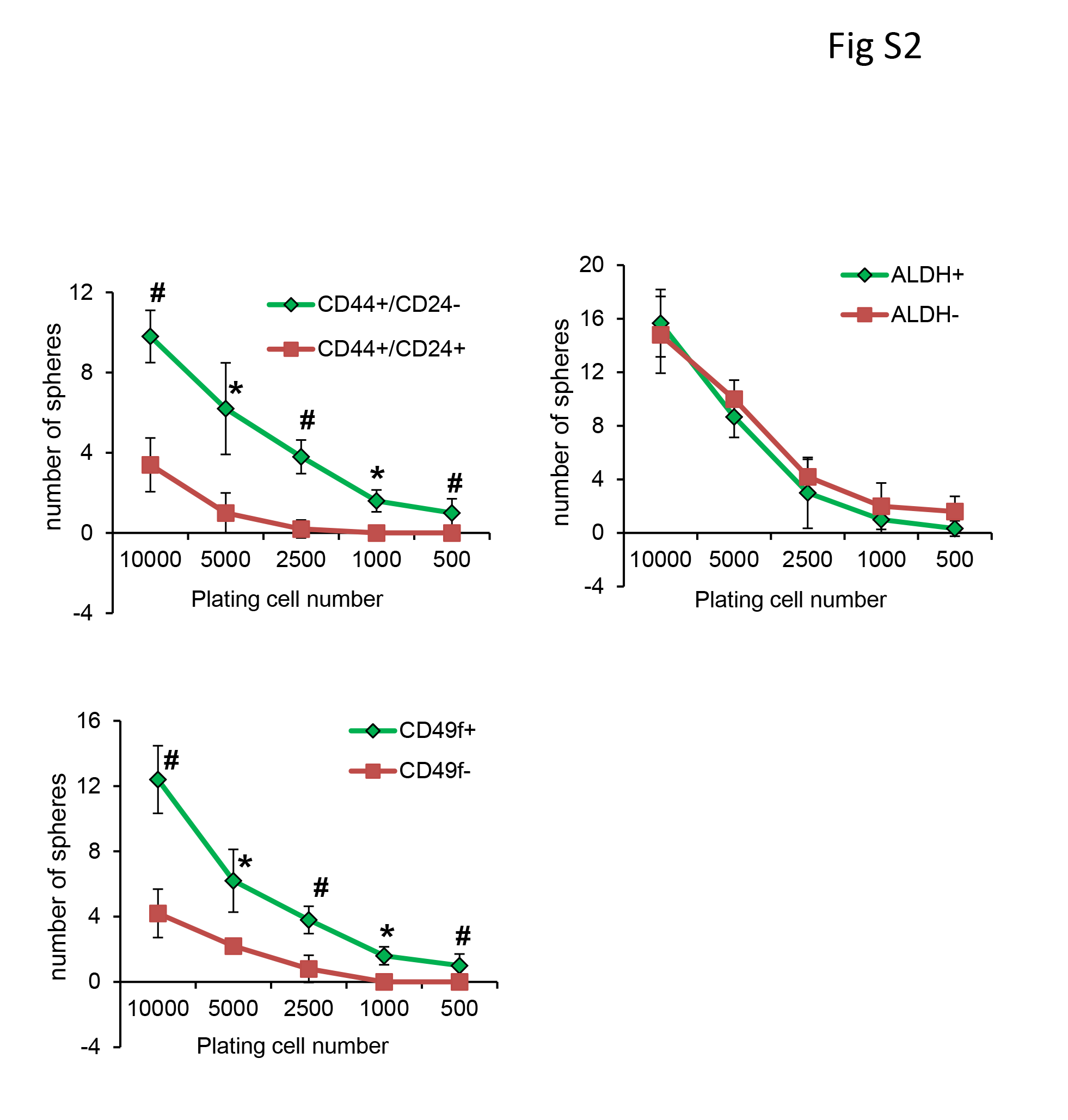

Supplement: S2 Fig — MCF10A cells were stained with CD44-PE/CD24-FITC, CD49f-FITC/EpCAM-PE, or ALDEFLUOR. The positive and negative cells were isolated by FACS sorting. The sorted MCF10A cells were subjected to a serial dilution and mammosphere formation assays. Each group was performed in triplicate and the number of spheres was counted and plotted as mean ± SD. #: p<0.001. *: p<0.05. (TIF) [file pone.0131285.s003.tif]

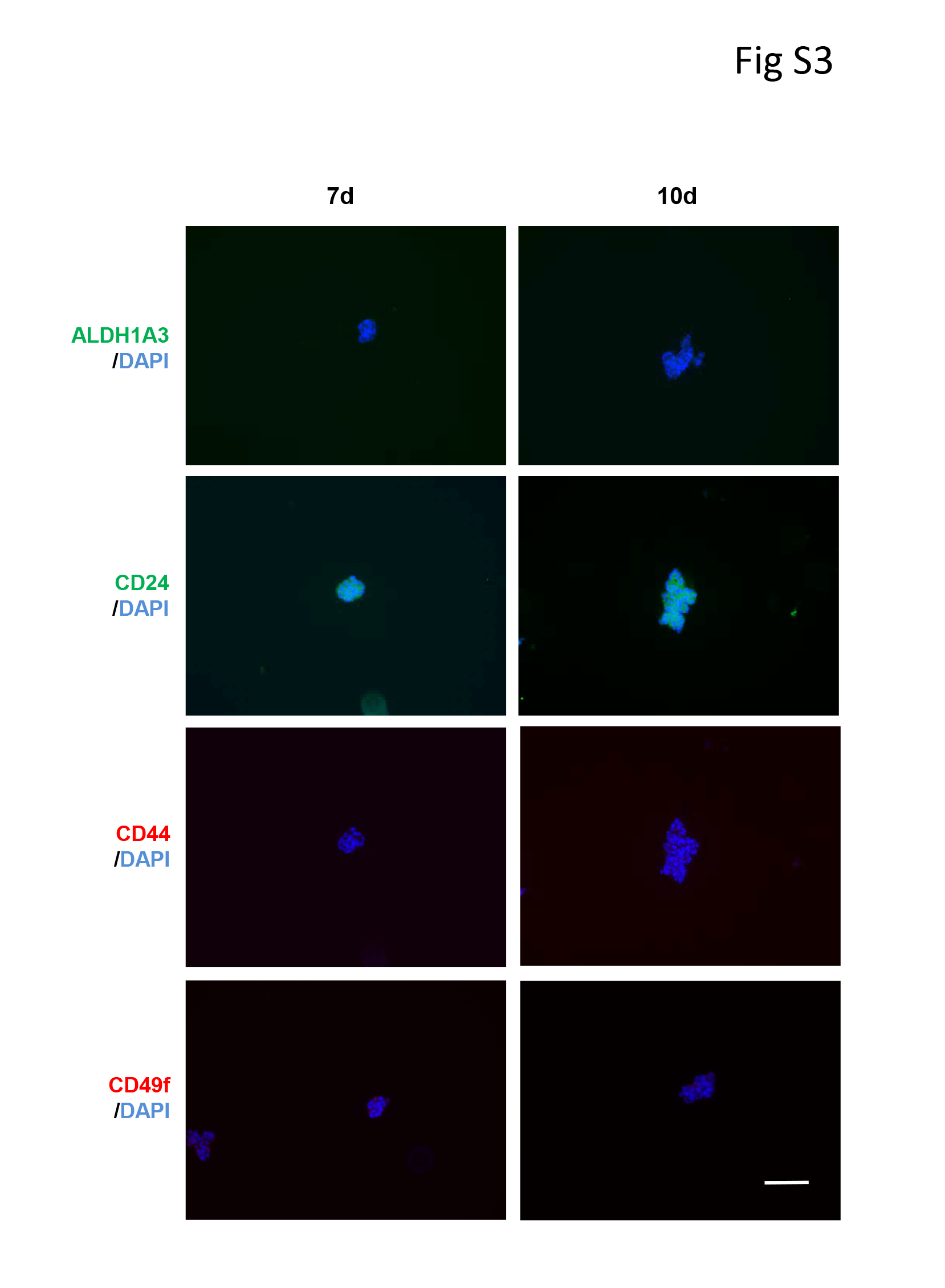

Supplement: S3 Fig — MCF10A cells were cultured using “on-top” of Matrigel method. 3D cultures were fixed, embedded and cut into sections. Expression of stem/progenitor markers were analyzed by immunofluorescence staining. Secondary antibodies were either AF488 (green)—or AF594 (red)—conjugated. DAPI was used for nuclear staining. Bars: 50μm. Original magnification: ×200. (TIF) [file pone.0131285.s004.tif]

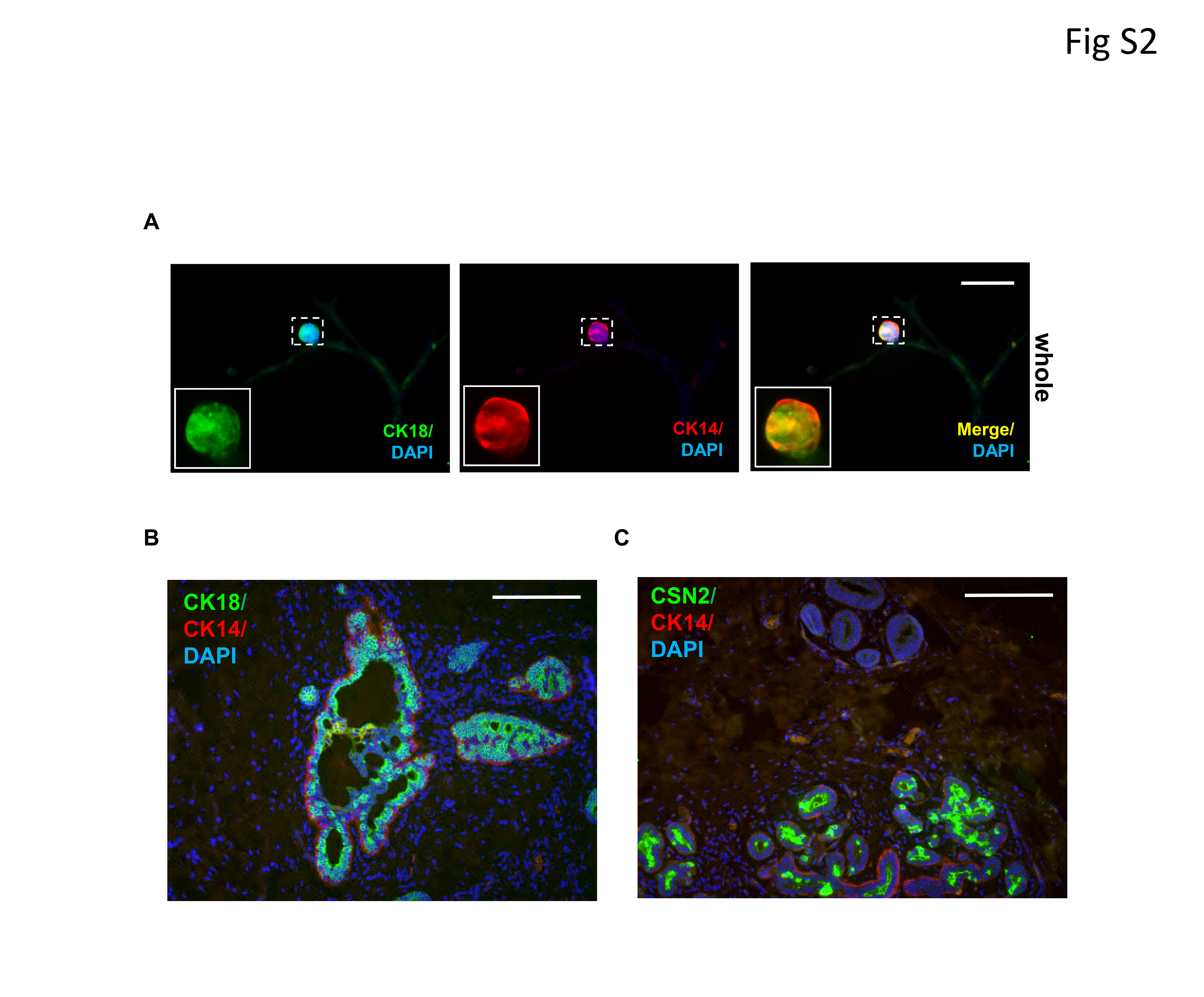

Supplement: S4 Fig — (A) Expression of CK18 (luminal) and CK14 (basal) in MCF10A formed acini. Enlarged views (white squares) of the indicated area (white dash squares) are shown. (B) CK14/CK18 double staining showed CK14+ basal layer and CK18+ luminal layer. (C) CSN2/CK14 double staining in normal human breast tissue. Secondary antibodies were either AF488 (green)—or AF594 (red)—conjugated. DAPI was used for nuclear staining. Bars: 100μm. Original magnification: ×200. (TIF) [file pone.0131285.s005.tif]
