# Supplementary material for: Evaluation of MCF10A as a Reliable Model for Normal Human Mammary Epithelial Cells
Source: PLoS One. 2015 Jul 6;10(7):e0131285. doi: 10.1371/journal.pone.0131285 (PMC4493126; doi:10.1371/journal.pone.0131285)
Supplement: S1 Table — (DOCX) [file pone.0131285.s006.docx]

**S1 Table.** **The primary antibodies used in this study**

| Primary antibody | Marker for | Source | Dilution |
| --- | --- | --- | --- |
| CK7 | luminal | Biocare | 1:200 |
| ER | luminal | Biocare | 1:100 |
| PR | luminal | Biocare | 1:100 |
| CK8 | luminal | Abcam | 1:200 |
| CK18 | luminal | Thermo | 1:200 |
| CK17 | myoepithelial | Biocare | Pre-diluted |
| CK5 | myoepithelial | Biocare | 1:200 |
| CK14 | myoepithelial | Biocare | 1:200 |
| P63 | myoepithelial | Biocare | 1:200 |
| α-SMA | myoepithelial | Biocare | 1:200 |
| vimentin | myoepithelial | Biocare | 1:200 |
| E-cadherin | Cell-cell junction | BD | 1:100 |
| N-cadherin | Cell-cell junction | BD | 1:100 |
| EpCAM | epithelial | Abcam | 1:200 |
| Mucin 1 | Differentiated epithelial | Abcam | 1:200 |
| CD49f | Stem/progenitor | R&D | 1:200 |
| ALDH1A3 | Stem/progenitor | Santa Cruz | 1:200 |
| Oct4 | Stemness | Stemcell | 1:200 |
| Sox2 | Stemness | Stemcell | 1:200 |
| Nanog | Stemness | Stemcell | 1:200 |
| CSN2 | Milk protein | Sigma | 1:350 |
| LALBA | Milk protein | Sigma | 1:2500 |
| CD44 | Stem/progenitor | Novus | 1:500 |
| CD24 | Differentiation | Biocare | 1:100 |
