# Supplementary material for: Evaluation of MCF10A as a Reliable Model for Normal Human Mammary Epithelial Cells
Source: PLoS One. 2015 Jul 6;10(7):e0131285. doi: 10.1371/journal.pone.0131285 (PMC4493126; doi:10.1371/journal.pone.0131285)
Supplement: S2 Table — Data represent the average positive cell percentage calculated from 10 viewing fields (original magnification, ×200). (DOCX) [file pone.0131285.s007.docx]

**S2 Table. Percentage of MCF10A cells expressing basal, luminal or breast-specific markers in 2D culture**

|  |  | + (%) | SD (%) |
| --- | --- | --- | --- |
| Basal | vimentin | 94.67 | 3.51 |
|  | SMA | 74.83 | 5.62 |
|  | N-cad | 94.17 | 3.55 |
|  | CK5 | 47.30 | 8.03 |
|  | CK17 | 10.63 | 2.57 |
|  | F-actin | 90.33 | 5.51 |
|  | P63 | 0.00 | 0.00 |
|  | CK14 | 0.50 | 0.30 |
| Luminal | CK18 | 3.86 | 1.91 |
|  | Muc1 | 5.67 | 2.52 |
|  | CK7 | 8.33 | 2.08 |
|  | CK8 | 46.79 | 8.02 |
|  | E-cad | 69.10 | 3.84 |
| Breast-specific | CSN2 | 0.00 | 0.00 |
|  | LALBA | 0.00 | 0.00 |

Data represent the average positive cell percentage calculated from 10 viewing fields (original magnification, ×200).
