# Supplementary material for: Evaluation of MCF10A as a Reliable Model for Normal Human Mammary Epithelial Cells
Source: PLoS One. 2015 Jul 6;10(7):e0131285. doi: 10.1371/journal.pone.0131285 (PMC4493126; doi:10.1371/journal.pone.0131285)
Supplement: S3 Table — Data represent the average positive cell percentage calculated from 10 viewing fields (original magnification, ×200). (DOCX) [file pone.0131285.s008.docx]

**S3 Table. Percentage of MCF10A cells expressing stem/progenitor markers in 2D culture**

|  | % | SD (%) |
| --- | --- | --- |
| EpCAM+/Muc1- | 64.63 | 9.31 |
| ALDH1A3+/CD49f+ | 28.33 | 4.16 |
| CD44+/CD24- | 84.67 | 9.07 |
| Nanog+ | 0.00 | 0.00 |
| Oct4+ | 92.3 | 3.32 |
| Sox2+ | 81.6 | 2.61 |

Data represent the average positive cell percentage calculated from 10 viewing fields (original magnification, ×200).
