# Supplementary material for: Evaluation of MCF10A as a Reliable Model for Normal Human Mammary Epithelial Cells
Source: PLoS One. 2015 Jul 6;10(7):e0131285. doi: 10.1371/journal.pone.0131285 (PMC4493126; doi:10.1371/journal.pone.0131285)
Supplement: S4 Table — Data represent the average positive cell percentage calculated from 10 viewing field in a thin section (original magnification, ×200). (DOCX) [file pone.0131285.s009.docx]

**S4 Table. Percentage of MCF10A cells expressing indicated markers in mammospheres**

|  | % | SD (%) |
| --- | --- | --- |
| CD49f | 2.67 | 2.52 |
| EpCAM | 89.37 | 3.10 |
| Muc1 | 67.67 | 4.93 |
| CD24 | 87.33 | 1.53 |
| CD44 | 21.67 | 2.08 |
| ALDH1A3 | 12.33 | 3.21 |
| CK5 | 79.67 | 11.06 |
| CK17 | 45.67 | 6.43 |
| CK8 | 92.33 | 2.52 |
| CK18 | 92.67 | 3.51 |
| CK7 | 18.33 | 4.16 |
| CSN2 | 54.67 | 6.11 |
| LALBA | 59.33 | 3.79 |

Data represent the average positive cell percentage calculated from 10 viewing field in a thin section (original magnification, ×200).
